# New U-Pb constraints and geochemistry of the East Kirkton Quarry, Scotland: Implications for early tetrapod evolution in the Carboniferous

**DOI:** 10.1371/journal.pone.0321714

**Published:** 2025-04-16

**Authors:** Hector K. Garza, Elizabeth J. Catlos, Thomas J. Lapen, Julia A. Clarke, Michael E. Brookfield

**Affiliations:** 1 Department of Earth and Planetary Sciences, The University of Texas at Austin, Austin, Texas, United States of America; 2 Center for Planetary Systems Habitability, The University of Texas at Austin, Austin, Texas, United States of America; 3 Department of Earth and Atmospheric Sciences, University of Houston, Houston, Texas, United States of America; Chinese Academy of Sciences, CHINA

## Abstract

The transition of vertebrates from aquatic to terrestrial environments during the late Devonian to early Carboniferous marks a crucial evolutionary milestone. However, this transition remains poorly understood due to a scarcity of early tetrapod fossils during the late Devonian to early Mississippian, creating a gap in the fossil record known as Romer’s Gap (~360–345 Ma). Recent discoveries have narrowed this gap, providing critical insights into early tetrapod evolution. The East Kirkton Quarry in Scotland’s Midland Valley, has yielded tetrapod fossils considered early stem amphibians and amniotes. They have been proposed to be Mississippian (early Carboniferous) in age, yet data to inform their precise ages remain limited. Here, zircon grains from two tuffaceous clastic limestones and shales were dated using Laser Ablation-Inductively Couple Plasma-Mass Spectrometry (LA-ICP-MS). The study presents detrital zircon U-Pb dates, which refine the current biostratigraphy ages assigned to *Westlothiana lizziae*, *Silvanerpeton miripedes*, *Balanerpeton woodi*, *Ophiderpeton kirktonense*, *Eucritta melanolimnetes*, and *Kirktonecta milnerae* to a maximum depositional age (MDA) of 341 ± 3 Ma (±2σ, n= 7 dates), placing them in the middle-lower Visean (Holkerian-Arundian) rather than the previous assigned upper Visean (Brigantian). This revised maximum depositional age places the East Kirkton Quarry fossils within the older, critical interval of Romer’s Gap, bridging a significant evolutionary time interval in the Mississippian fossil record, and allows for refining future tetrapod time trees. X-ray Fluorescence and X-ray Diffraction analyses reveal heterogeneity in the lower East Kirkton Limestone of the East Kirkton Quarry, with variations in elemental and mineralogical compositions, reflecting episodic volcanic and detrital inputs and hydrothermal activity.

## Introduction

**Fig 1:**
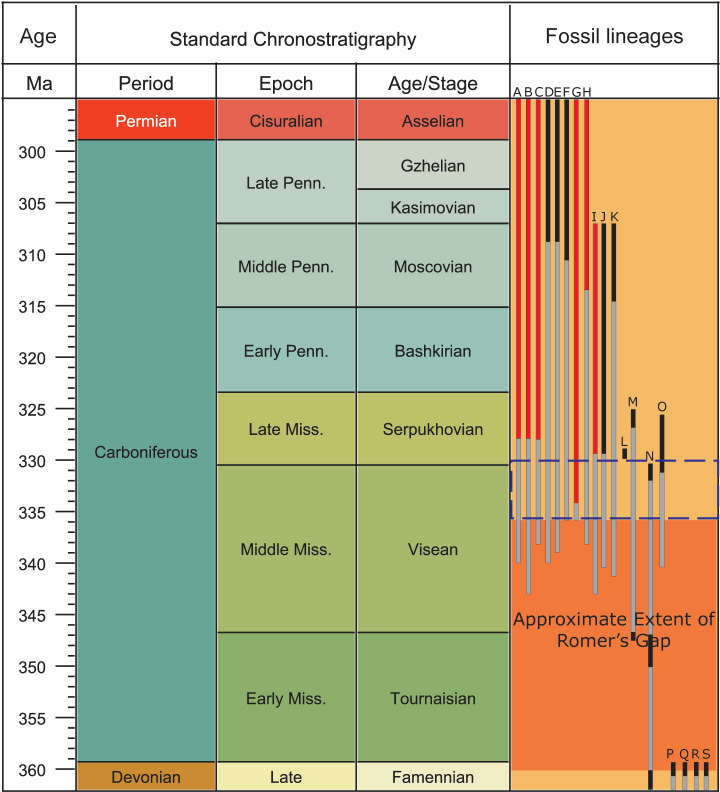
Geological time scale showing tetrapod taxa distribution and fossil lineages (modified from [3,7,8]). Colors indicate fossil occurrences and phylogenetic ghost lineages: red represents tetrapod fossils from the East Kirkton Quarry; black denotes tetrapod fossils from other localities; and gray indicates phylogenetic tree-modeled ghost lineages. Taxa abbreviations: A, Anthracosauria; B, Temnospondyli; C, Microsauria; D, Seymouriamorpha; E, Diadectidae; F, Nectridea; G, Aistopoda; H, Amniota; I, Baphetidae; J, Colosteidae; K, Gephyrostegidae; L, *Casineria*; M, *Crassigyrinus*; N, Whatcheeriidae; O, Adelogyrinidae; P, *Ventastega*; Q, *Ichthyostega*; R, *Acanthostega*; S, *Tulerpeton*. The approximate extent of Romer’s Gap is highlighted by the dark orange box. The blue dashed box indicates the current biostratigraphic age range assigned to fossils from the East Kirkton Limestone (based on [3,9–12]).

The transition of vertebrates from aquatic to terrestrial environments represents one of the most pivotal evolutionary milestones in Earth’s history. This transition, spanning from the late Devonian to early Carboniferous (Tournaisian and Visean stages), was marked by significant anatomical and physiological adaptations that enabled life to thrive on land [1–3]. However, our understanding of this evolutionary leap has been hindered by the scarcity of early tetrapod and vertebrate fossils from the late Devonian to the early Mississippian, complicating efforts to trace the development of terrestrial adaptations in both stem and crown group lineages [3,4]. Fig 1 shows a gap in the early terrestrial tetrapod fossil record, known as Romer’s Gap, spanning from the late Devonian (Famennian age, ~360 Ma) to the middle Mississippian (Visean age, ~336 Ma) [1,5–8]. Recent fossil discoveries, however, have refined the duration of Romer’s Gap to approximately 15 million years, extending from the late Devonian (Famennian age, ~360 Ma) to the early Mississippian (Tournaisian age, ~345 Ma) [1,3–5,8]. Recent discoveries have begun to illuminate this enigmatic period, shedding light on the evolutionary innovations that facilitated colonization of terrestrial environments.

Romer’s Gap remains one of the most intriguing and enigmatic intervals in paleontology, as it obscures our understanding of a critical period in vertebrate evolution. The exact causes of Romer’s Gap are not fully understood but are frequently attributed to sampling and taphonomic biases in the Carboniferous tetrapod fossil record [3,8,13–16]. According to [3], coal mining has differentially facilitated the discovery of tetrapod fossils in sediments associated with coal beds, creating a sampling bias in the fossil record [17]. This taphonomic bias arises because coal beds preferentially preserve tetrapods that inhabited low-energy environments, such as coal swamps [3,18,19]. In addition to sampling and taphonomic explanations, other theories have been proposed to account for Romer’s Gap. Some researchers suggest that environmental changes, such as climate cooling associated with the End-Devonian Mass Extinction or low atmospheric oxygen levels during the early Carboniferous, may have constrained the diversity and abundance of early terrestrial vertebrates, thereby contributing to the gap in the fossil record [8,15,20–23]. However, other studies contribute Romer’s Gap as primarily results of sampling bias leading to an incomplete fossil record, rather than being driven by environmental or atmospheric factors such as climate change or oxygen levels [8,15,24]. This ongoing debate underscores the complexity of understanding Romer’s Gap and highlights the need for continued exploration and discovery to illuminate this formative chapter in vertebrate evolution.

**Fig 2:**
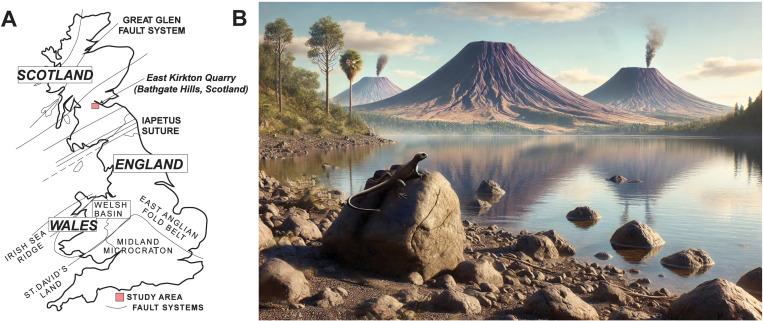
(A) Map of the United Kingdom highlighting the East Kirkton Quarry study area (red) in the Bathgate Hills, Midland Valley of Scotland (modified from [41]). (B) Generalized environmental reconstruction of the East Kirkton setting, based on previous schematic diagrams and interpretations (modified from [34,42–44]). The reconstruction includes *Westlothiana lizziae* (not to scale) depicted resting on a rock.

Fossil sites such as the East Kirkton Quarry in the Bathgate Hills of Scotland’s Midland Valley have yielded numerous stem and crown tetrapod fossils, including microsaurs, baphetids, aistropods, temnospondyls, and anthracosaurs. Fig 2a shows the location of the site, which has produced *Westlothiana lizziae*, classified as a tetrapod and a potential early amniote [3,9,25–33]. We include a generalized reconstruction of the East Kirkton Quarry and *Westlothiana lizziae* during the Mississippian in Fig 2b. These discoveries are filling critical gaps in evolutionary history and providing insights into how early tetrapods adapted and survived during this enigmatic period. Each new find offers valuable evidence, helping to piece together the evolutionary transition of life from aquatic to terrestrial environments [8,31,34–37]. However, the precise age of these fossils remains approximate, as it has primarily been determined through biostratigraphy and correlated to regional substage ages [3,9–12,38–40]. Previous studies in the Bathgate Hills using conodont, plant macrofossil, and palynomorph biostratigraphy have constrained the age of the East Kirkton Limestone tetrapods to the Visean (Asbian substage) at approximately 333.5–335.5 Ma [3,9,12,38,40]. Alternatively, other studies have assigned the East Kirkton Quarry tetrapod fossils to the younger Visean (Brigantian substage), with an estimated age of 330–330.9 Ma [6,10,11,33,39]. These varying age estimates highlight the need for further refinement in dating to enhance our understanding of these critical fossils.

Acquiring accurate and precise age constraints are essential for understanding the timing of locomotor shifts and evolution of key traits that enabled early tetrapods to transition between aquatic and terrestrial environments [8,12,15,31,35–37,45]. In this study, we present comprehensive data from analysis of eleven samples utilizing mineralogical X-ray diffraction (XRD), elemental X-ray fluorescence (XRF), and the first detrital zircon U-Pb Laser Ablation-Inductively Coupled Plasma-Mass Spectrometry (LA-ICP-MS) from two samples constraining the East Kirkton Quarry terrestrial tetrapods. We establish a maximum depositional age (MDA) for the tuffaceous clastic limestones and shales bearing the early Carboniferous tetrapods, previously dated solely via biostratigraphy. The refined U-Pb MDA provides critical temporal constraints on the emergence of early stem amniotes and stem amphibia, offering new insights into the evolutionary history of early tetrapods and contributing to efforts to fill Romer’s Gap.

### Geologic background

The East Kirkton Quarry, located in the Bathgate Hills of Scotland’s Midland Valley, is an exceptional paleontological site. It provides one of the most comprehensive successions of the earliest known and most diverse terrestrial tetrapod fauna from the Carboniferous with a previously estimated Visean age of ~335 Ma based on biostratigraphy correlations and paleobotany including macrofossils and spore microfossils (Figs 1, 2a) [3,9,33,42,46]. This fossil-bearing East Kirkton Limestone, a formation composed of heterogeneous interbedded sedimentary and volcanic rocks comprised of limestone, shale, and volcaniclastic deposits including clastic limestone, tuffaceous limestone, and tuffs [33,43,46–50]. These lithologies were deposited in a restricted, mineral-rich, toxic lake fed by marine incursions and hot springs, located approximately 8 km from active volcanoes. This setting facilitated exceptional fossil preservation [10,33,42,43,46,49–54].

The Midland Valley of Scotland has been described as a tropical forest ecosystem supporting diverse faunal and plant communities, including tetrapods, arthropods, and gymnosperm-pteridosperm plants [9,42,55]. The East Kirkton Limestone has yielded seven distinct tetrapod fossils of varying sizes and morphologies. These include *Westlothiana lizziae* [9,25,27], the anthracosaur-like *Silvanerpeton miripedes* [9,56,57], *Eldeceeon rolfei* [9,58,59], the temnospondyl *Balanerpeton woodi* [9,60], the aistopod *Ophiderpeton kirktonense* [9,61], the baphetid-like *Eucritta melanolimnetes* [9,28,62], and the putative microsaur *Kirktonecta milnerae* [9,33,62,63]. These taxa are considered stem tetrapods, with some (*Westlothiana lizziae*, *Silvanerpeton miripedes*, and *Eldeceeon rolfei*) identified as stem amniotes and others (*Balanerpeton woodi*) as Lissamphibia, providing key insights and serving as a minimum age estimate during early tetrapod evolution [9,27,28,30,33].

**Fig 3:**
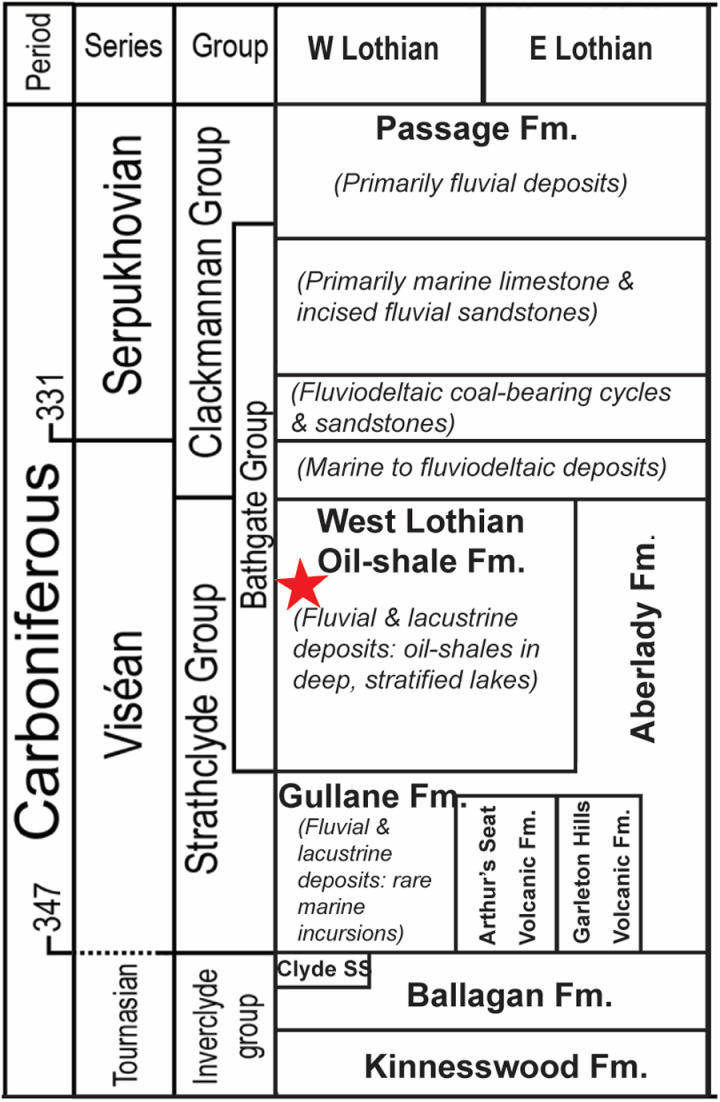
Generalized geological formations and chronostratigraphic divisions of the West and East Lothian localities in Scotland, modified from [49,67]. The East Kirkton Limestone at the East Kirkton Quarry is highlighted with a red star.

**Fig 4:**
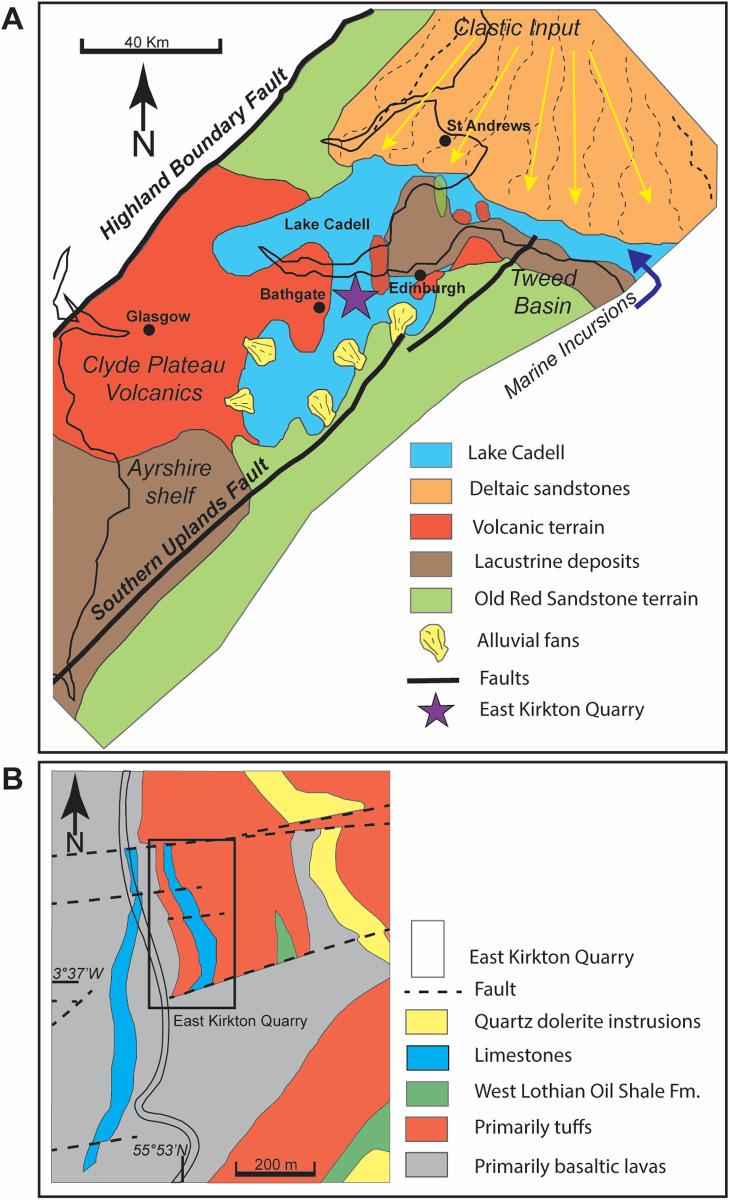
(A) Paleoenvironmental reconstruction of the East Kirkton Quarry locality and surrounding areas, illustrating a unique restricted hydrothermal lacustrine setting influenced by sporadic marine incursions. The diagram highlights various volcanic and clastic deposits near Lake Cadell, modified from [49,68]. Reprinted from [49] under a CC BY license, with permission from Elsevier, original copyright 2017. (B) Geologic map of the East Kirkton Quarry showing sample collection locality by [46] and modified from [50,68]. Reprinted from [50] under a CC BY license, with permission from Elsevier, original copyright 2017.

Fig 3 shows the general stratigraphy of the East Kirkton Quarry, which has been described extensively by [42,64], and more recently and in detail by [43,44,46]. The East Kirkton Limestone is part of the West Lothian Oil Shale Formation within the Strathclyde Group. At the East Kikrton Quarry, the stratigraphic sequence comprises three members: the East Kirkton Limestone, the overlying Little Cliff Shale, and the Geikie Tuff [46,49,50,65–67]. The Geikie Tuff is represented by a 4.20 meters thick member with samples collected by [46] through a numeric system encompassing units 1–31. The Little Cliff Shale is a ~2-meter-thick member with samples collected from units 32–36, while the East Kirkton Limestone is a ~ 9-meter-thick member with an unexposed base in the quarry with samples collected from units 37–88 [46]. These units contain a mix of lacustrine, marine, and volcaniclastic deposits, reflecting episodic volcanic activity and sporadic marine incursions in proximity to the depositional environment (Fig 2b) [42,46,49,68]. Multiple studies concur that the Strathclyde Group, including the East Kirkton Limestone Member, represents a paleoenvironmental setting of volcanic, alkaline, mineral-rich lacustrine system, sustained by hot springs and punctuated by sporadic marine incursions (Fig 4a) [10,42,46,49,50,54,55,69,70]. Fig 4 displays the paleoenvironmental reconstruction and geologic map of the East Kirkton Quarry locality along with the sediment sources for the lacustrine depositional environment.

In the East Kirkton Limestone, tetrapod fossils are described from Units 76 and 82, with tuffs interbedded throughout the stratigraphic section [42,46]. This study focuses on East Kirkton Limestone and, specifically, on Units 66, 69, 72, 76, 80, 81, 82, 83, 84, 87, and 88, following the stratigraphic sampling nomenclature of [46]. Unit 66 consists of a 105–140 mm thick thinly laminated limestone containing distorted chert bands, stromatolitic structures, tuffaceous deposits (where an amphibian jaw was discovered), and a basal shale facies [46]. Unit 69 features thinly laminated limestone with chert bands overlying coarse-grained tuffaceous clastic limestone, yielding marine arthropod fossils such as a centrum and the head shield of *Hibbertopertus* [46]. Unit 72 is marked by coarse-grained calcareous tuff with variable grain sizes, including large limestone fragments, tuffs from other beds, and pyroclastic volcanic material [46]. Unit 76, a 200–350 mm thick calcareous tuff, contains carbonate spherulites and is underlain by spherulitic laminated limestone and shale. Fossils from this unit include a scorpion, eurypterid cuticle, bone fragments, and the terrestrial tetrapod *Eldeceeon rolfei* [33,46,58,59]. Unit 80 comprises a thin bed of calcareous tuff with ostracod fossils and stromatolitic limestone, grading upward into laminated shale and containing a myriapod fossil [46].

Unit 81, with a thickness of 80–140 mm, exhibits lateral variability in lithology and includes tuffaceous clastic limestone and laminated shale containing ostracods, volcanic fragments, coal, and plant remains [46]. Unit 82 is a composite unit divided into three layers: the top layer features 1 mm thick bituminous shale overlying interbedded calcareous tuffs and black shales with ostracods, the middle layer consists of 80–200 mm thick tuffaceous clastic limestone, and the lower layer includes a 10–70 mm thick calcareous tuff with carbonate spherulites, a 20 mm thick tuff, and a 10 mm thick bituminous shale. Fossils from Unit 82 include stem tetrapods including *Westlothiana lizziae*, *Silvanerpeton miripedes*, *Balanerpeton woodi*, *Ophiderpeton kirktonense*, *Eucritta melanolimnetes*, and *Kirktonecta milnerae* [9,28,46,56,57,62]. Unit 83 consists of shale with coalified plant remains overlying a massive spherulitic tuffaceous clastic limestone containing volcanogenic fragments, with large Lissamphibia bones recovered from this unit [46]. Unit 84 includes a basal thin carbonate-spherulitic laminated shale, overlain by a thin spherulitic tuffaceous clastic limestone, followed by spherulitic black shale and coarse-grained spherulitic tuffaceous clastic limestone [46]. Units 87 and 88 are both characterized by large coarse-grained tuffaceous clastic limestones containing rip-up clasts of laminated limestone, tuffaceous calcareous shale, carbonate spherulites, and volcanic fragments, with Unit 88 grading upward into a shaly tuffaceous calcareous sandstone [46].

The East Kirkton Quarry locality has undergone extensive tectonism. This region is bound by two major fault systems: the Southern Upland Fault and the Highland Boundary Fault [42]. During the Carboniferous, the West Lothian Shale, including the East Kirkton Limestone members, was deposited in response to rapid extensional tectonics associated with a north-south trending fault system exhibiting a right-lateral strike-slip component. This tectonic regime produced half-grabens, resulting in the formation of semi-closed continental basins [3,42,49,50,66,67,71]. These intracratonic basins were subsequently filled with deltaic, fluvial, and lacustrine deposits [49,67]. The rifting also triggered localized, transient calc-alkaline basaltic volcanic activity. This is evidenced by the limited lateral continuity of the tuffs and the pyroclastic fragments containing pseudomorphs of olivine and plagioclase phenocrysts, which indicate close proximity between volcanic centers and the deposition of the East Kirkton Limestone [10,44,50,66,72].

The East Kirkton Limestone was deposited in a lacustrine environment within a low-lying topographic depression, as evidenced by low-energy laminated sediments and the presence of freshwater biota throughout the sequence [10,42,46,49,50]. This Carboniferous body of water, referred to as Lake Cadell, experienced periodic disruption from episodic volcanic eruptions, resulting in the deposition of ash and tuff layers of varying thicknesses, ranging from 5 cm to 1 m [10,42,49,51]. These volcanic deposits were interbedded as volcaniclastic rocks throughout the East Kirkton Limestone, suggesting that the lake was short-lived, persisting for only a few tens of thousands of years, and its existence closely coincided with volcanic activity in the region (Fig 4b) [42,50].

During the 1800s, early studies identified volcanogenic sediments associated with the East Kirkton Limestone and interpreted the tuffs as ashfall deposits resulting from episodic volcanic eruptions [44,47,51,69]. The volcanic activity responsible for these deposits was attributed to small basaltic cinder cone volcanoes located approximately 8 km north of the quarry (Fig 2b, 4a). These eruptions produced pyroclastic and lava flows, contributing to the sedimentary record of the region [10,42,44,51,55]. More recent research, however, has revised this interpretation. The tuffs and volcanogenic sediments of the East Kirkton Limestone are now understood to represent secondary pyroclastic or epiclastic deposits. These were formed through the erosion and subaqueous redeposition of volcanic materials and associated fossils into Lake Cadell, where the limestone was accumulating [42,44,65]. The volcanic sediments were introduced to the lake through episodic epiclastic pulses triggered by basaltic eruptions, which occurred every few hundred to a few thousand years. These sediments were transported from volcanic flanks to the lake by debris flows and lahars [42,44,65,73]. Evidence for subaqueous deposition includes graded bedding observed at several horizons and the presence of marine ostracod fossils [42,44,49,65].

Volcanism in the region significantly impacted the aquatic ecosystem of Lake Cadell by altering the lake’s temperature and increasing its toxicity, which likely affected the aquatic biota [42]. Evidence of the lake’s hot spring environment is found in the precipitated lacustrine limestone, which is characterized by abundant carbonate spherulitic growths [10,42,46,49,69]. The stratigraphic sequence of the East Kirkton Limestone reveals distinct lithological transitions, including volcaniclastic sediments, tuffaceous carbonates, and lacustrine shales, interbedded with marine shales containing marine fossils such as ostracods and fish. These transitions provide evidence of sporadic marine incursions into the lake, which periodically disrupted the lacustrine environment [42,46,49,50,54].

According to [55], the alkali volcanism that occurred during the Carboniferous in Scotland’s Midland Valley represents one of the earliest, if not the first, documented examples of this type of volcanism globally. This basaltic volcanism, coupled with the region’s equatorial, tropical forest and humid environment, created conditions that likely facilitated evolutionary innovations. These environmental factors may have played a crucial role in enabling the transition of tetrapods from aquatic to terrestrial habitats [10,42,55]. However, the volcanic activity may have also triggered forest fires, potentially driving terrestrial fauna toward nearby water bodies such as Lake Cadell. This behavior has been proposed to explain the remarkable preservation of tetrapod fossils within the lake’s sediments [42].

## Methods

### Sample collection and information

In this study we dated two East Kirkton Limestone samples (EK82 and EK83) composed of mixture lithologies including tuffaceous clastic limestones and shales, with sample EK82 derived from the matrix hosting the stem tetrapod fossils (Fig 1) [3,9,33,42]. We also acquired XRF and XRD analyses from eleven samples (EK66, EK69, EK72, EK76, EK80, EK81, EK82, EK83, EK84, EK87, and EK88) composed of tuffaceous limestones and shales with volcaniclastic sediment input. Sample EK76 contained the terrestrial tetrapod *Eldeceeon rolfei* (specimen NMS G. 1986.39.1), while sample EK82 contained *Westlothiana lizziae* (specimen NMS G. 1990.72.1), *Silvanerpeton miripedes* (specimen UMZC T1317), *Balanerpeton woodi* (specimen GLAHM V2051), *Ophiderpeton kirktonense* (specimen NMS G 1988.3.1), *Eucritta melanolimnetes* (specimens UMZC T1347; UMZC T1348), and *Kirktonecta milnerae* (specimens UMZC 2002a; UMZC 2002b) [9,25,27,28,56–63]. All eleven samples in this study are sourced from the Lower East Kirkton Limestone, situated in the East Kirkton Quarry in the Bathgate Hills of Scotland. The East Kirkton Quarry is a Site of Special Scientific Interest (SSSI) in Scotland with restrictions on sample collection due to its paleontological significance, thus the samples analyzed in this study ranging from 200 to 500 grams were provided by the National Museum of Scotland and come directly from the detailed study by [46]. All necessary permits for this study were secured as a research material loan from the National Museum of Scotland’s Department of Natural Sciences: Paleobiology, with authorization from Dr. Andrew J. Ross, ensuring full compliance with all relevant regulations.

### U-Pb geochronology

Traditional heavy mineral separation techniques were used following methods by [41]. A total of 58 zircon grains from sample EK82 and 12 zircon grains from sample EK83 were mounted in epoxy and inspected with secondary electron (SE) and color cathodoluminescence (CL) imaging using a Hitachi SU-8700 field emission scanning electron microscope (SEM) at the Scanning Electron Microscope Laboratory in the Bureau of Economic Geology at UT Austin. However, due to the limited number of zircons in each sample, some grains were analyzed twice, thus yielding a total of 82 U-Pb dates for sample EK82, and 20 U-Pb dates for sample EK83. Following imaging, the zircon grains were dated using an Analytik Jena PlasmaQuant MS Elite quadrupole LA-ICP-MS at the University of Houston. The instrument uses a Photon Machine Excite (193 nm) ArF laser ablation system and obtains isotopic measurements using ion counting. A dry ablated aerosol is introduced to the instrument by pure He carrier gas containing the desired isotopic analytes, which for this study consists of ^201^Hg, ^202^Hg, ^204^Hg+^204^Pb, ^206^Pb, ^207^Pb, ^208^Pb, ^232^Th, ^235^U and ^238^U. Each analysis consists of a 2-pulse cleaning ablation, a background measurement taken with the laser off, a 30 second measurement with the laser firing and a 30 second washout. Zircon grains were analyzed with a laser beam 20 µm in diameter and with 240 shots at an 8 Hz repetition rate. Inter- and intra-elemental isotopic fractionation of Pb and Pb/U isotopes were corrected using primary and secondary zircon standards with known ages (Plesovice and FC5z) [74,75]. The typical ratio of unknown standards measurements was 5:1. Systematic uncertainties resulting from calibration corrections are usually 1–2% for ^206^Pb/^207^Pb and ^206^Pb/^238^U. Fully propagated errors utilized for U-Pb zircon dates. U-Pb analyses are included as (Supplementary file (Table S1)).

Statistical values, MDAs, and concordia diagrams were produced by IsoplotR, Densityplotter and detritalPy with a bandwidth value of eight [76–78]. A ^206^Pb/^238^U vs ^207^Pb/^235^U and ^206^Pb/^238^U vs ^206^Pb/^207^Pb 10% discordance filter was implemented for all LA-ICP-MS zircon dates. The youngest detrital zircon date (Dz YSG) is obtained from the youngest zircon date [79]. The youngest single grain (YSG) MDA approach is calculated from the most concordant youngest zircon date [80]. The youngest cluster of 3+ grains (YC2σ+3) is determined by computing the weighted mean of the youngest zircon grain cluster consisting of three or more grains at 2σ uncertainty [80]. Moreover, the youngest mode weighted mean (YMWM) was calculated following the method outlined by [81]. This involved using LA-ICP-MS zircon dates that constituted the youngest age mode from a KDE peak, calculating a weighted mean of more than three grains overlapping at 2σ uncertainty, with an approximate MSWD of 1 [41,82].

### Whole-rock mineralogy and geochemistry

In addition to the zircon dates, whole rock mineralogical (XRD data) were obtained from all eleven samples at the GeoMatCI facility at UT Austin. Whole rock samples were manually homogenized, ground, and sieved to a 250 µm mesh size. XRD analyses were performed using a Bruker D8 instrument equipped with Cu Kα radiation and a nickel filter, along with a LYNXEYE solid-state detector. The analyses were carried out at a voltage of 45 kV and a current of 40 mA, employing a 2θscan axis ranging from 3° to 70°, with step increments of.0195° (2θ) and a duration of 1 s per step. Whole rock X-ray patterns were determined through Rietveld refinement utilizing Bruker TOPAS 4.2 software.

Whole rock elemental compositions were obtained using a portable x-ray fluorescence (XRF) instrument at the University of Texas at Austin following the methods of [83]. Samples were grounded for homogenization, pressed into a pellet, and analyzed using a Bruker S1 Titan 800 ED-XRF (portable XRF) instrument equipped with Rh x-ray tube. Analyses consisted of 15 Kv excitation voltage for major element oxides (Na2O, MgO, Al2O3, SiO2, P2O5, K2O, CaO, TiO2, MnO and Fe2O3 for 30 seconds, and 50 Kv excitation voltage for trace elements (S, V, Cr, Co, Ni, Cu, Zn, Ga, As, Rb, Sr, Y, Zr, Nb, Mo, Ba, Pb, Th and U) for 60 seconds. Detection limits for individual majors and trace elements are included as (Supplementary file (Table S2)). XRF analyses were calibrated modifying the Bruker MudRock Air calibration and generating our own clastic rock standards. Our references consist of five international commercially available accepted standards (SBC-1, SGR-1b, SCo-2, ShBOQ-1, and SRM 70b), and five siltstone/sandstone internal standards [82]. The major elements average uncertainty is ± 1 Wt.%, while trace element average uncertainty is ± 30 ppm. The calibration comparison is included as (Supplementary file (Table S3)). To evaluate trace element enrichment, an enrichment factor (EF) was calculated by normalizing each element to Al and comparing their ratios to the upper continental crust composition [84], following the methodology of [85]. An EF < 1 indicates depletion relative to the upper continental crust, an EF ≈ 1 suggests similar concentrations, and an EF > 1 indicates enrichment. Sediment provenance discrimination diagram plotted after [86].

## Results

### Zircon U-Pb dates

**Fig 5:**
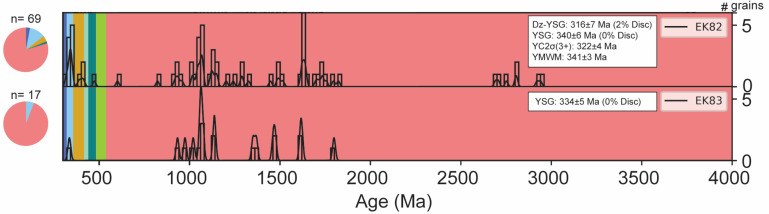
Samples EK82 and EK83 kernel density estimation (KDE) distribution plots. LA-ICP-MS ^238^U-^206^Pb and ^207^Pb-^206^Pb zircon dates with (±2σ) uncertainties from 10% discordance filter dataset. Maximum depositional age (MDA) methods (detrital youngest single grain [Dz YSG]; most concordant youngest single grain [YSG]; youngest cluster of three or more grains overlapping within 2σ uncertainty [YC2σ+3]; youngest mode weighted mean [YMWM]). Colors represent the period (pink: Precambrian; light green: Cambrian; teal: Ordovician; aquamarine: Silurian; orange: Devonian; light blue: Mississippian; blue: Pennsylvanian).

**Fig 6:**
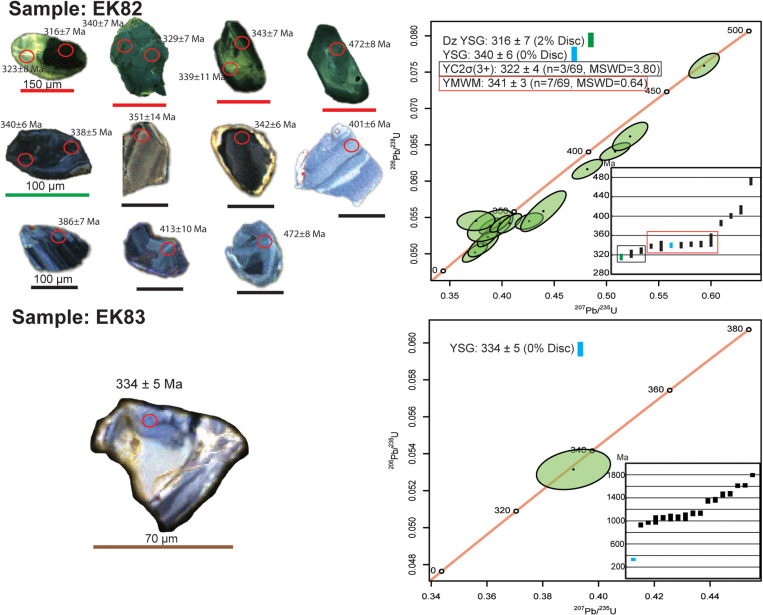
Cathodoluminescence (CL) images and concordia diagrams of representative zircon grains from samples EK82 and EK83. CL images display ^238^U-^206^Pb LA-ICP-MS dates, while concordia diagrams show ^238^U-^206^Pb data, filtered to include only grains with ≤10% discordance for zircons younger than 1000 Ma. Zircon grain distributions illustrate ^238^U-^206^Pb Paleozoic dates for sample EK82 and combined ^238^U-^206^Pb and ^207^Pb-^206^Pb Precambrian–Paleozoic dates for sample EK83. Maximum depositional age (MDA) approaches are color-coded as follows: green for the youngest detrital single grain (Dz YSG), light blue for the most concordant youngest single grain (YSG), black box for the youngest cluster of three or more grains overlapping within 2σ uncertainty (YC2σ+3), and red box for the youngest mode weighted mean (YMWM). All uncertainties are reported as 2σ.

Fig 5 displays a KDE plot of 69 zircon dates from sample EK82 after applying a ≤10% discordance filter. These dates range from the Precambrian to the Pennsylvanian and are used to estimate an MDA for Unit 82, as described by [46] (Supplementary Table S1). Of the 69 zircon dates, one is Pennsylvanian, two are Pennsylvanian-Mississippian, six are Mississippian, one is Mississippian-Devonian, two are Devonian, one is Devonian-Silurian, one is Ordovician, and 55 are Precambrian. The youngest MDA estimates include a detrital youngest single grain (Dz YSG) date of 316 ± 7 Ma (2% discordance) and the most concordant youngest single grain (YSG) date of 340 ± 6 Ma (0% discordance). Fig 6 presents concordia diagrams that demonstrate the concordance of zircon grains between the ^238^U- ^206^Pb and ^235^U-^207^Pb chronometers. The figure also highlights representative grain morphologies and U-Pb ages of the zircon grains analyzed. The observed morphologies include rounded, anhedral, and subhedral forms, reflecting characteristics typical of both detrital and volcanic zircon grains (Fig 6). The YC2σ (3+) approach yields an age of 322 ± 4 Ma (n = 3, MSWD = 3.80), while the youngest mode weighted mean (YMWM) provides an age of 341 ± 3 Ma (n = 7, MSWD = 0.64, bandwidth = 8) (Figs 5,6).

Sample EK83, located stratigraphically half a meter below EK82 yielded 17 zircon dates after applying a ≤10% discordance filter. The dates range from the Precambrian to the Pennsylvanian. These dates were used to estimate an MDA for Unit 83, as described by [46] (Fig. 5; Supplementary Table S1). Of the 17 dates, only one is Mississippian, while the remaining 16 are Precambrian. The youngest MDA estimate for EK83 is the most concordant youngest single grain (YSG) date of 334 ± 5 Ma (0% discordance). However, due to the lack of additional Mississippian-aged grains, no further MDA calculations using other approaches could be performed (Figs 5,6).

### East Kirkton Limestone Mineralogy (XRD) analyses

Mineralogical analysis (XRD) of the eleven samples analyzed in this study (EK66, EK69, EK72, EK76, EK80, EK81, EK82, EK83, EK84, EK87, and EK88) reveals variations in mineralogical composition across the dataset. Detailed weight percent quantifications for each sample are provided in Table 1. On average, the samples are primarily composed of calcite (63 wt.%), followed by quartz (11 wt.%), clay minerals including illite/mica+illite/smectite (9 wt.%), chlorite (5 wt.%), and kaolinite (~0.5 wt.%). Additionally, the samples contain dolomite (7 wt.%), K-feldspar (11 wt.%), and plagioclase (1 wt.%) on average.

**Table 1. X:** ray diffraction (XRD) mineral composition (Wt.%) from the East Kirkton Limestone.

Sample	Quartz	K-spar	Plagioclase	Calcite	Dolomite	Chlorite	Kaolinite	Illite/Mica+Illite/Smectite	Total Wt. %
EK66	24	3	0	53	11	3	0	6	100
EK69	24	4	0	54	7	4	0	7	100
EK72	3	1	4	71	2	8	0	11	100
EK76	11	2	0	63	10	4	1	9	100
EK80	3	3	1	74	2	6	1	10	100
EK81	20	3	0	67	5	0	1	4	100
EK82	4	5	4	39	4	14	2	28	100
EK83	4	1	0	85	4	2	0	4	100
EK84	26	11	0	32	6	7	1	17	100
EK87	7	2	0	73	10	2	0	6	100
EK88	3	2	0	80	11	2	0	2	100
Avg.	11	3	1	63	7	5	.5	9	
Max	26	11	4	85	11	14	2	28	
Min	3	1	0	32	2	0	0	2	

### East Kirkton Limestone Major and Trace (XRF) Geochemistry analyses

Elemental (XRF) analyses of the eleven samples from the East Kirkton Limestone reveal compositions consistent with heterogeneous limestones, cherts, and shales, all mixed with clastic and volcaniclastic sediments. Detailed weight percent quantification values are provided in Table 2. The average concentrations of major element oxides are dominated by CaO (28.6 wt.%), followed by SiO₂ (26.4 wt.%), Fe₂O₃ (6.5 wt.%), Al₂O₃ (5.4 wt.%), MgO (2.7 wt.%), Na₂O (2.2 wt.%), TiO₂ (1.5 wt.%), K₂O (0.6 wt.%), P₂O₅ (0.4 wt.%), and MnO (0.1 wt.%).

**Table 2:** XRF major-element oxide concentrations (Wt.%) from the East Kirkton Limestone.

Sample	Na_2_O	MgO	Al_2_O_3_	SiO_2_	P_2_O_5_	K_2_O	CaO	TiO_2_	MnO	Fe_2_O_3_
**EK66**	2.1	2.8	3.6	35.8	0.4	0	26.0	0.7	0.1	6.1
**EK69**	2.1	3.0	4.6	38.5	0.4	0.2	23.2	0.9	0.1	7.0
**EK72**	2.3	3.3	7.4	22.3	0.6	0.7	27.4	2.8	0.1	7.7
**EK76**	2.0	3.3	5.0	25.8	0.5	0.4	28.9	1.1	0.1	6.6
**EK80**	2.3	2.6	7.1	22.1	0.4	0.8	29.8	1.9	0.1	7.2
**EK81**	2.5	0.9	1.0	25.1	0.5	0	37.1	0.1	0.2	3.2
**EK82**	1.8	5.0	12.5	36.2	0.6	1.8	11.9	4.7	0.1	13.1
**EK83**	2.3	1.4	2.9	13.5	0.2	0.2	40.6	0.6	0.1	3.1
**EK84**	2.2	3.3	9.0	41.1	1.9	1.9	12.8	2.1	0.1	9.7
**EK87**	2.3	2.5	3.3	16.2	0.4	0.4	37.7	0.7	0.1	4.1
**EK88**	2.7	2.1	3.2	14.2	0.3	0.3	39.6	0.6	0.1	4.0
**Avg.**	2.2	2.7	5.4	26.4	0.4	0.6	28.6	1.5	0.1	6.5
**Max**	2.7	5.0	12.5	41.1	1.9	1.9	40.6	4.7	0.2	13.1
**Min**	1.8	0.9	1.0	13.5	0.2	0	11.9	0.1	0.1	3.1

**Fig 7:**
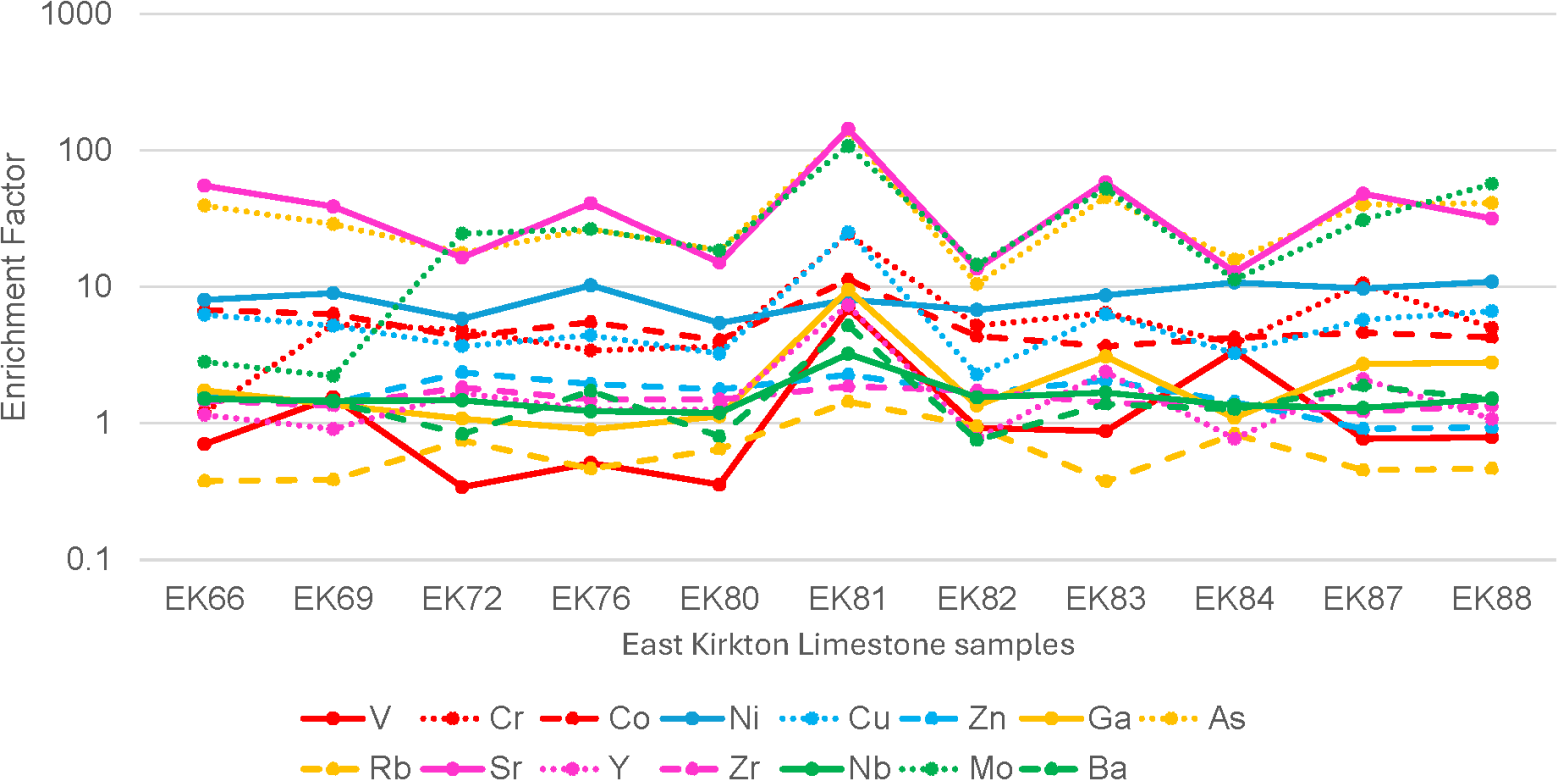
Trace element enrichment factor (EF) of samples in this study from the East Kirkton Quarry Limestone after [85]. An EF < 1 indicates depletion relative to the upper continental crust, an EF ≈ 1 suggests similar concentrations, and an EF > 1 indicates enrichment.

Concentrations for each trace element are detailed in Table 3. Trace element analyses indicate average concentrations dominated by Sr (3470 ppm), followed by S (2435 ppm), Ba (241 ppm), Zr (103 ppm), Cr (63 ppm), Ni (58 ppm), Zn (42 ppm), Cu (39 ppm), Rb (27 ppm), V (26 ppm), Co (17 ppm), As (13 ppm), Nb (13 ppm), Mo (12 ppm), Y (10 ppm), and Ga (10 ppm). The average enriched trace element concentration in the eleven samples (EF > 1) include V (1.60), Cr (6.73), Co (5.41), Ni (8.51), Cu (6.57), Zn (1.67), Ga (2.44), As (38.60), Sr (43.19), Y (1.88), Zr (1.51), Nb (1.59), Mo (31.71), and Ba (1.66). These results are illustrated in Fig 7.

**Table 3:** XRF trace-element concentrations (ppm) from the East Kirkton Limestone.

Sample	S	V	Cr	Co	Ni	Cu	Zn	Ga	As	Rb	Sr	Y	Zr	Nb	Mo	Ba
EK66	1971	10	10	16	38	37	27	7	14	10	4565	6	66	9	1	195
EK69	2355	28	56	19	54	39	30	7	13	13	4079	6	77	11	1	237
EK72	1748	10	83	21	57	45	82	9	13	41	2805	18	170	18	18	224
EK76	2499	10	39	18	67	36	45	5	13	17	4672	9	93	10	13	310
EK80	1911	10	60	19	51	38	59	9	13	34	2477	13	133	14	13	207
EK81	2146	26	53	7	10	39	10	10	13	10	3113	10	22	5	10	177
EK82	2113	46	150	36	112	47	97	19	13	88	3941	14	272	32	18	343
EK83	2018	10	43	7	33	30	28	10	13	8	3884	10	51	8	15	145
EK84	4914	120	79	25	127	48	60	11	14	55	2647	10	153	20	10	410
EK87	2467	10	80	10	42	31	14	10	13	11	3644	10	50	7	10	224
EK88	2464	10	37	9	46	35	14	10	13	11	2339	5	54	8	18	175
Avg.	2435	26	63	17	58	39	42	10	13	27	3470	10	103	13	12	241
Max	4914	120	150	36	127	48	97	19	14	88	4672	18	272	32	18	410
Min	1748	10	10	7	10	30	10	5	13	8	2339	5	22	5	1	145

## Discussion

### Age of East Kirkton limestone quarry tetrapods

The Mississippian tetrapod fossil record is predominantly dated and understood through conodont biostratigraphy, palynostratigraphy, and plant macrofossil biostratigraphy, which collectively serve as the foundation for calibrating phylogenetic models (Fig 1) [3,9,13,33,42]. However, reliance on biostratigraphy alone for dating fossils from the East Kirkton Quarry poses significant challenges. Conodont biostratigraphy, for instance, is based on marine environments, whereas the East Kirkton Limestone represents a non-marine sedimentary unit within the Bathgate Hills, limiting the applicability of this method at this site [42].

Reassessments of Carboniferous tetrapod ages previously determined by biostratigraphy further illustrate these limitations. For example, the tetrapod fauna from Joggins, Nova Scotia, originally dated to the Duckmantian age based on plant macrofossil biostratigraphy [87], was later revised to the middle-late Langsettian using palynostratigraphy [3,88,89]. Similarly, the baphetid tetrapod *Spathicephalus* from East Fife, Scotland, was initially assigned to the late Mississippian (Serpukhovian stage, Pendleian age) using palaeobotanical biostratigraphy but was subsequently redated to the middle Mississippian (Viséan stage, Asbian age) following refined stratigraphic analyses [3,7]. These cases underscore the inherent limitations of palaeobotanical biostratigraphy and emphasize the need for more precise geochronological approaches.

Robust U-Pb radiometric dating is critical for accurately determining these units and the ages of tetrapod groups, including early stem amphibians and stem amniotes, from the East Kirkton locality. Furthermore, phylogenetic trees calibrated exclusively with regional biostratigraphic data risk incorporating biases from these data. The Brigantian substage, which provides the biostratigraphic framework for the East Kirkton Limestone tetrapods, is primarily used in European contexts. Attempts to correlate the Brigantian with equivalent stages in North America have revealed biostratigraphic age inconsistencies in both regions [90–92]. Age estimates for the Brigantian vary significantly: the British Geological Survey assigns it an age of 330–330.9 Ma, while other European studies suggest ranges of 327 ± 2.5 Ma to 334 ± 7 Ma or 329–331.5 Ma [93,94].

Additionally, prior studies have suggested different ages for the East Kirkton Quarry tetrapods, ranging from an Asbian substage age of 333.5–335.5 Ma to a Brigantian age of 330–330.9 Ma [6,9–12,33,38–40]. These uncertainties highlight the need for a more robust and precise dating framework, particularly given the incomplete Mississippian tetrapod fossil record that contributes to Romer’s Gap (Fig 1). In this study, we establish MDAs using detrital zircon U-Pb dates obtained through LA-ICP-MS analyses of samples EK82 and EK83 (units 82 and 83) of the East Kirkton Quarry.

For samples EK82 and EK83, the East Kirkton Limestone exhibits a combination of volcanic and detrital inputs, as shown by the kernel density estimation (KDE) plots of U-Pb zircon dates (Fig 5). The multimodal distribution of zircon dates, ranging from the Carboniferous to the Precambrian, indicates a diverse provenance involving contributions from rock units of varying ages and/or the reworking of older sedimentary deposits. Consequently, the zircon dates from these samples are not interpreted as zircon crystallization ages but are instead considered MDAs (Figs 5, 6). MDAs derived from detrital zircons are governed by the “law of detrital zircon,” a principle stating that a geological formation cannot be older than its youngest constituent zircon grain but may be younger [41,82,95–98]. This principle establishes a threshold age for MDAs.

In this study, MDAs for sample EK82 were determined using four methods: the youngest detrital single grain (Dz YSG), the most concordant youngest single grain (YSG), the youngest cluster of three or more grains overlapping at two sigma (YC2σ (3+)), and the youngest mode weighted mean (YMWM) age (Figs 5, 6). For sample EK83, only the YSG method was employed, as the sample contained a single U-Pb Mississippian-age zircon, with the remaining grains being Precambrian in age. The results for sample EK82 reveal U-Pb dates younger than the previously assigned Brigantian regional substage age of 330–330.9 Ma. Specifically, sample EK82 yielded a Dz YSG age of 316 ± 7 Ma (2% discordance) and a YC2σ (3+) age of 322 ± 4 Ma (n = 3, MSWD = 3.80). It is important to note that the Dz YSG and YC2σ (3+) methods are often considered less conservative, as they rely on the youngest individual zircons or clusters. These approaches may produce ages significantly younger than the true depositional age (TDA) due to potential lead (Pb) loss or systematic uncertainties [41,82,96,99–101]. Such limitations underscore the need for careful interpretation of MDAs when constraining the depositional age of sedimentary units.

The most concordant youngest single grain (YSG) ages for samples EK82 and EK83 overlap within 2σ uncertainty (Fig 6). Sample EK82 yields a YSG age of 340 ± 6 Ma (0% discordance), while sample EK83 produces a YSG age of 334 ± 5 Ma (0% discordance). The youngest mode weighted mean (YMWM) age from sample EK82, calculated at 341 ± 3 Ma (n = 7, MSWD = 0.64), also overlaps within 2σ uncertainty with the YSG ages of both samples. However, these MDAs are older than the Brigantian regional substage age of 330–330.9 Ma. The YSG age from EK82 suggests a regional Visean (Asbian-Holkerian) age for the unit, whereas the YMWM places it in the Visean (Holkerian-Arundian) age. For EK83, the YSG age corresponds to a Visean (Pendleian-Holkerian) age. Nonetheless, the reliability of the MDA for EK83 is limited due to its reliance on a single Mississippian age zircon amidst a predominantly Precambrian zircon population (Figs 5, 6). Consequently, the MDA for EK83 serves only as supplementary support for the more robust U-Pb MDA derived from EK82. The concordia diagram in Fig 6 evaluates the age consistency between the two U-Pb chronometers, ^238^U- ^206^Pb and ^235^U-^207^Pb, while assessing potential disturbances in the U-Pb system, such as Pb loss. The diagram reveals a prominent age cluster at approximately 340 Ma for sample EK82, which is consistent within uncertainty with the youngest mode weighted mean (YMWM) age of 341 ± 3 Ma. However, it also identifies several U-Pb dates younger than the YMWM age and the previously assigned Brigantian regional substage age of 330–330.9 Ma. These younger dates suggest potential Pb loss or other post-depositional processes that may have affected some zircon grains (Fig 6).

Using our U-Pb zircon dates and the YMWM method, we refine the age of Unit 82 (sample EK82) from the East Kirkton Quarry to 341 ± 3 Ma, with the possibility of a younger MDA based on zircon grains yielding Pennsylvanian-Mississippian dates. However, it is important to acknowledge discrepancies in previously assigned ages for the East Kirkton Quarry tetrapods. While biostratigraphic studies have suggested ages ranging from Brigantian to Asbian (330–335.5 Ma) [6,9–12,33,38–40], the National Museum of Scotland has traditionally assigned an older Chadian age (345 Ma) for public communication regarding the age of *Westlothiana lizziae*, recovered from sample EK82. This Chadian assignment is approximately one million years older than the upper uncertainty limit of our U-Pb MDA of 341 ± 3 Ma, highlighting the need for further refinement and correlation of biostratigraphic and radiometric dating methods. *(A. Ross and S. Walsh, personal communication, December 13, 2024, and January 10, 2025.)*

**Fig 8:**
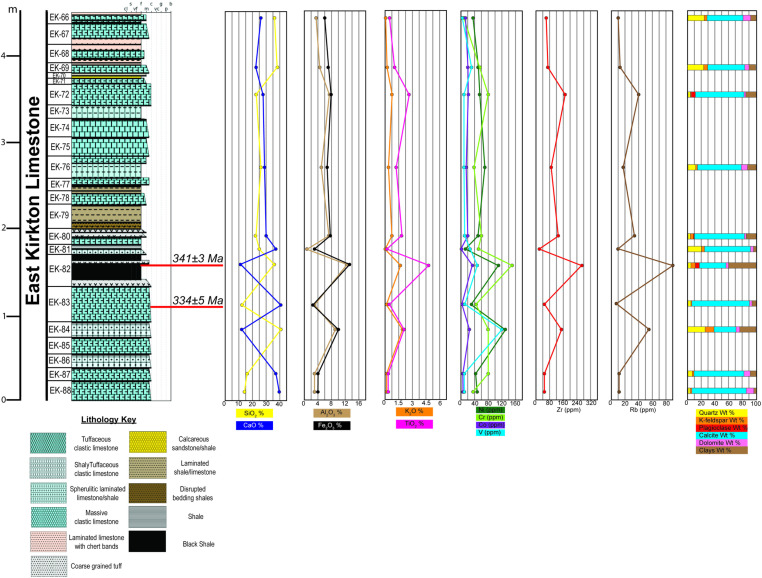
Stratigraphic, chronostratigraphic, XRD, and XRF chemostratigraphic plots for Units 66–88 of the East Kirkton Limestone at the East Kirkton Quarry locality, incorporating LA-ICP-MS YMWM dates obtained from this study’s samples EK82 and EK83 alongside the stratigraphic column adapted from [46]. The chemostratigraphic data include proxies such as %SiO₂, representing quartz, clastic sediment input, and chert; %CaO, indicating carbonate content; %Al₂O₃, reflecting clay minerals; %Fe₂O₃, associated with clays and carbonates; %K₂O, indicative of K-feldspar, mica, and illite/clay content; %TiO₂, representing continentally derived sediment; and [ppm Ni, Cr, Co, V], proxies for basaltic and mafic sediment input. Additionally, ppm Zr serves as a proxy for continentally derived sediment and volcanic phases, while ppm Rb indicates contributions from K-feldspar, mica, and illite/clays. Quantitative XRD data provide whole-rock mineralogy percentages, offering a detailed geochemical and mineralogical characterization of the East Kirkton Limestone.

**Fig 9:**
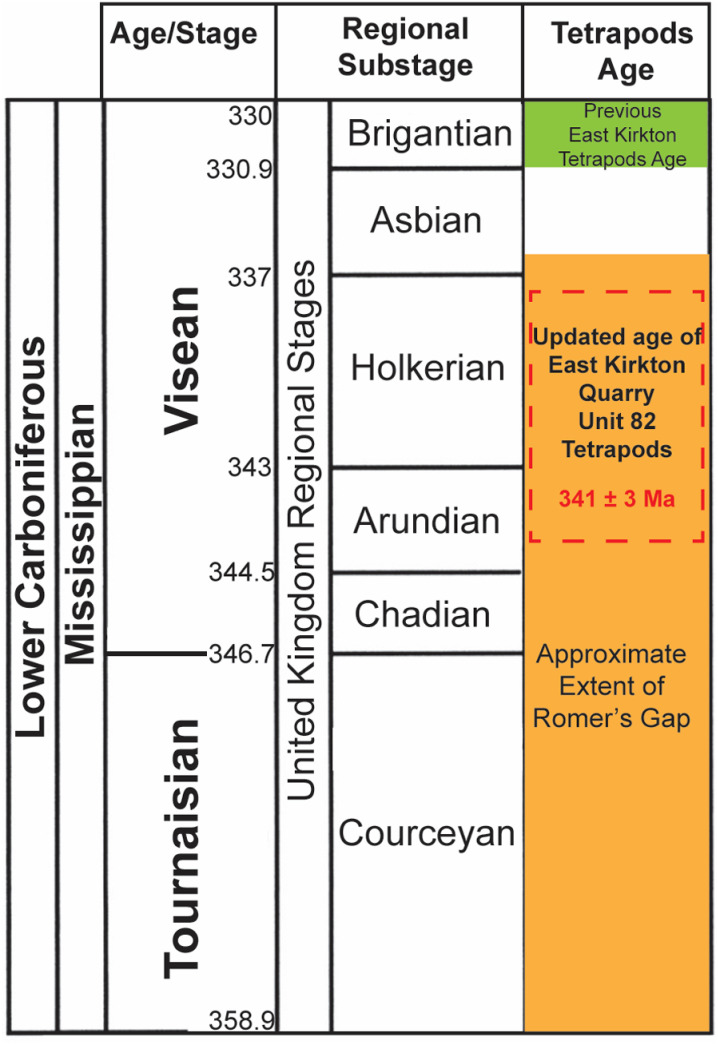
Mississippian geological time scale illustrating the previously assessed biostratigraphic age of the East Kirkton Quarry tetrapods (green box) and the updated U-Pb interpreted age from this study (red dashed box) [9,28,46,56,57,62]. The approximate temporal extent of Romer’s Gap is highlighted in the orange box. Regional substages and age references are based on the British Geological Survey geological time chart.

Fig 8 presents a stratigraphic column adapted from [46], incorporating this study’s interpreted MDAs, as well as chemostratigraphic and mineralogical trends observed in the East Kirkton Limestone at the East Kirkton Quarry. Fig 9 shows the updated age shift of *Westlothiana lizziae*, *Silvanerpeton miripedes*, *Balanerpeton woodi*, *Ophiderpeton kirktonense*, *Eucritta melanolimnetes*, and *Kirktonecta milnerae* hosted in sample EK82 from the upper Visean (Brigantian) to the middle-lower Visean (Holkerian-Arundian). As a result, these tetrapod fossils are now positioned within the temporal extent of Romer’s Gap, contributing valuable data to help bridge this critical interval in the fossil record. The refined maximum depositional ages from this study are pivotal not only for understanding the timing of evolutionary milestones, such as the transition of tetrapods from aquatic to terrestrial environments, but also for calibrating phylogenetic models. These improved age constraints play a vital role in estimating divergence times for early terrestrial tetrapod lineages, thereby enhancing our understanding of their evolutionary history.

### East Kirkton limestone age limitations and considerations

While this study provides a geochronological framework for the East Kirkton Quarry using detrital zircon U-Pb dating, several limitations should be acknowledged. The use of maximum depositional ages (MDAs) derived from detrital zircons presents inherent challenges. Although these ages establish a maximum depositional age for Unit EK82 of the East Kirkton Quarry, they only constrain the upper limit, meaning the true depositional age could be younger. Additionally, several factors can introduce uncertainties in U-Pb LA-ICP-MS dating, including lead (Pb) loss, isotopic ratio measurement uncertainties, isotope fractionation relative to external standards and their associated age uncertainties, and the presence of common Pb [41,97,102,103].

Another consideration in detrital zircon geochronology is the notion that at least 117 zircon grains should be dated to establish reliable MDAs. This threshold, widely recognized in the geochronology community, primarily applies to clastic sedimentary rocks such as sandstones and siltstones [104]. However, the East Kirkton Limestone differs significantly from typical detrital clastic lithologies. The unit consists of a mixture of basaltic tuffaceous clastic limestones and shales, with continuous influence from basaltic volcanic debris flows that bias the depositional environment. Consequently, the samples analyzed in this study must be evaluated differently from traditional clastic sedimentary rocks.

Furthermore, constraints imposed by the Site of Special Scientific Interest (SSSI) designation at the East Kirkton Quarry limit both access to the site and the volume of sample material available for analysis. As a result, this study was restricted to a minimal sample size (200–500 gram per sample) provided by the National Museum of Scotland, which did not yield sufficient zircon grains to meet the 117-grain threshold per sample. Despite this limitation, sample EK82 provided meaningful and interpretable distribution of U-Pb zircon dates, including an abundance of Carboniferous-aged grains, supporting its utility in constraining the depositional age of the unit.

Despite the basaltic volcaniclastic nature of the East Kirkton Limestone, this study highlights the role of felsic clastic input in providing detrital zircons suitable for radiometric dating. Basaltic volcanism typically lacks zircon due to its low felsic content, particularly in silicon (Si) and zirconium (Zr) [105–108]. However, the presence of felsic-derived detrital zircons within the East Kirkton Limestone enables the application of radiometric dating techniques that can refine and calibrate regional lacustrine biostratigraphy.

The most reliable MDAs obtained from U-Pb LA-ICP-MS analyses align closely with U-Pb chemical abrasion isotope dilution thermal ionization mass spectrometry (CA-ID-TIMS) dates when calculated using weighted averages rather than solely the youngest single grains. This approach mitigates potential biases from younger ages affected by Pb loss and older ages influenced by inherited zircon grains or cores [41,81,82,96]. CA-ID-TIMS remains the gold standard for U-Pb dating, providing unparalleled precision and accuracy with age uncertainties as low as ≤0.1% at 2σ [109–111]. This technique effectively minimizes the impact of Pb loss, ensuring more robust and reliable age constraints [41,81,96]. Given these considerations and the U-Pb zircon data from this study, the youngest mode weighted mean (YMWM) MDA method was determined to be the most appropriate approach. Previous studies have demonstrated that the YMWM method closely aligns with CA-ID-TIMS results, particularly for early Paleozoic zircon grains, as it avoids the inclusion of anomalously young grains affected by Pb loss and excessively old inherited grains or cores [41,81].

### East Kirkton limestone mineralogy

Previous studies on the mineralogy of the East Kirkton Quarry, based on hand samples, thin-section petrography, and semi-quantitative SEM and XRD analyses, have determined that the primary components of the East Kirkton Limestone are dominated by carbonate and silicate minerals and include calcite, dolomite, quartz, feldspar, serpentine, kaolinite, chlorite, illite/smectite, and trace amounts of pyrite [38,48,49,73].

In this study, we obtained quantitative mineralogical data via XRD analysis for the lower portion of the East Kirkton Limestone from the East Kirkton Quarry, focusing on samples EK66, EK69, EK72, EK76, EK80, EK81, EK82, EK83, EK84, EK87, and EK88. The results revealed a primary mineralogy comprising calcite, quartz, illite/mica + illite/smectite, dolomite, chlorite, K-feldspar, plagioclase, and minimal kaolinite (Table 1). Our findings are consistent with the mineralogy described in previous studies [10,49,50,73]. It is important to note that XRD analyses cannot quantify amorphous content, preventing the measurement of chert in our samples. Additionally, pyrite was not detected through whole-rock XRD mineralogical analysis due to its trace-level abundance. However, pyrite was identified during the zircon mineral separation process. Notably, the 4.5-meter sequence of the East Kirkton Limestone analyzed in this study exhibits significant mineralogical variation between individual samples (Fig 8). These variations, primarily reflected as fluctuations in calcite, quartz, and clay content, are likely the result of episodic clastic input into Lake Cadell and changes in the lake’s environment, alternating between a restricted hydrothermal lake and periods of sporadic marine incursions (Fig 4a) [42,46,49].

Carbonates within Lake Cadell formed due to high concentrations of dissolved calcium and bicarbonate ions, derived from hydrothermal fluids, which facilitated calcite precipitation [10,73]. However, other studies have proposed that the dissolved calcium in the lake derived through leaching from the volcanic terrain, or with the mixture between the lake and atmospheric carbon [50,112]. According to [73], clay minerals such as chlorite and kaolinite correlate to higher Al_2_O_3_ content, and reflect the terrigenous clastic detrital sediment transported to Lake Cadell. However, the hydrothermal origin of the lake environment could also have generated clay minerals such as chlorite, illite/mica, and illite/smectite without only being introduced as detrital sediment. These clay minerals can form through the alteration of primary minerals or volcanic material under the influence of hydrothermal fluids, elevated temperatures, and specific chemical conditions [113–116]. Chlorite typically forms in hydrothermal settings through the alteration of mafic minerals or volcanic glass in the presence of magnesium- and iron-rich hydrothermal fluids [117–119]. Illite/mica and illite/smectite form through the hydrothermal alteration of feldspars, volcanic glass, or smectite, with potassium-rich fluids playing a critical role in illite formation. In hydrothermal systems, smectite gradually transforms into illite with increasing temperature and time, involving the exchange of cations (e.g., K^+^ replacing Na^+^ or Ca^2+^) and the loss of interlayer water [120–122]. The restricted nature of Lake Cadell allowed carbonate deposition to dominate due to minimal siliciclastic input. Exceptions to this occurred during debris flows or marine incursions, which introduced siliciclastic material into some limestone units. These episodic events highlight the dynamic depositional environment of the East Kirkton Limestone.

### East Kirkton Limestone major and trace element geochemistry

The East Kirkton Limestone presents significant challenges for chemostratigraphic analysis due to its complex lithological composition, which includes limestone, shale, siltstone, sandstone, coal, and volcaniclastic sediments within single bedding units [43,44,46]. As a result, whole-rock geochemical analyses can be difficult to interpret because these rocks cannot be characterized chemostratigraphically in the same way as typical clastic rocks or carbonates. Previous studies using XRF major and trace element geochemistry have shown that the East Kirkton Limestone is primarily composed of CaO and SiO₂, with notable concentrations of Al₂O₃ and Fe₂O₃ [73,123]. These findings align with the XRF major element results presented in this study (Table 2, Fig 8). Notably, the XRF results indicate elevated sulfur concentrations in the eleven samples analyzed, ranging from 1748 ppm to 4914 ppm, which reflect hydrothermal activity in the paleo-lake environment.

In this study, the samples are divided into three groups based on elemental variability and trends: Group 1 includes samples with high major and trace element variability (EK80, EK81, EK82, EK83, EK84, EK87, EK88); Group 2 comprises samples with stable chemostratigraphic concentrations and trends (EK72, EK76); and Group 3 consists of samples with slight chemostratigraphic variability (EK66, EK69).

#### Group 1: High variability samples.

Group 1 samples exhibit significant variability in CaO and SiO₂ content, which is attributed to an increase in SiO₂ derived from terrigenous detrital sediments, corroborated by XRD analyses showing elevated quartz and/or clay minerals (Fig 8, Tables 1–2). Al₂O₃ and Fe₂O₃ concentrations also display variability, following consistent chemostratigraphic trends that correlate with clay content, as confirmed by the XRD results. The K₂O and Rb concentrations correspond with K-feldspar and clay mineral content, while TiO₂ and Zr concentrations also show consistent trends and serve as proxies for terrigenous sediment input. Samples EK82 and EK84 demonstrate the highest detrital sediment content, supported by XRD data showing elevated quartz and clay minerals. Trace elements such as Ni, Cr, Co, V, and Fe₂O₃ follow similar trends, reflecting mafic input (Fig 8, Table 3). This suggests that these samples contain detrital sediments derived from basaltic lavas, with EK82 and EK84 showing the highest concentrations of basaltic material.

#### Group 2: Stable concentration samples.

Group 2 samples, EK72 and EK76, show minimal variability in elemental concentrations. CaO and SiO₂ levels are stable, with SiO₂ contributions likely from hydrothermal fluids in addition to detrital sources (Fig 8, Table 2). XRD analyses reveal that these samples are dominated by calcite, with minor quartz and clay content (Fig 8, Table 1). Al₂O₃ and Fe₂O₃ concentrations are similar between the samples, correlating with clay mineral content. The K₂O and Rb concentrations follow identical trends, corresponding to clay minerals rather than K-feldspar, as indicated by XRD results. TiO₂ and Zr concentrations increase slightly from EK76 to EK72, reflecting an increase in detrital sediment input, corroborated by XRD data. While Ni, Cr, Co, and V concentrations are elevated, they are lower than in Group 1 (Fig 8, Table 3). EK72 shows a higher enrichment of trace elements, indicating a greater input of basaltic-derived sediment compared to EK76.

#### Group 3: Slight variability samples.

Group 3 samples, EK66 and EK69, exhibit higher SiO₂ concentrations than CaO, despite XRD results showing higher carbonate content (Fig 8, Tables 1–2). This discrepancy is explained by the presence of chert, which contributes SiO₂ detected by XRF but not quantified by XRD due to its amorphous nature. Fe₂O₃ concentrations are consistent across these samples, reflecting similar clay content. K₂O and Rb concentrations align with clay and K-feldspar content as shown by XRD analyses. TiO₂ and Zr concentrations display a slight decreasing trend from EK69 to EK66, indicating reduced detrital sediment input. Trace element concentrations (Ni, Cr, Co, V) are the lowest among the three groups, suggesting that the quartz and clay content in these samples originates from a non-mafic source (Fig 8, Tables 1, 3).

### Interpretation of geochemical results

Based on our XRF data, the results align with previous studies suggesting that elevated concentrations of trace elements, such as Ni and Cr—and in this study, Co and V—in the East Kirkton Limestone are attributable to the calc-alkaline composition of the lower Carboniferous lavas and tuffs in the Midland Valley of Scotland (Table 3) [73,123]. However, our XRF analyses indicate that the lower portion of the East Kirkton Limestone stratigraphic section in the quarry exhibits a greater enrichment of trace elements, likely due to the basaltic volcanism in the area [73]. suggests that volcaniclastic sediments transported to the lake were the primary source of major and trace elements, with the exception of SiO₂ and CaO, which were attributed to hydrothermal fluids circulating through the underlying Tyningham or Gullane Formations. However, not all SiO₂ can be attributed to hydrothermal sources; a portion is derived from detrital sediment, as evidenced by the presence of quartz and feldspars in the samples (Fig 8, Table 1). Additional SiO₂ contributions come from clay minerals, which could have been deposited as detrital sediments or generated in situ by hydrothermal activity (Fig 8). As discussed in the mineralogy section of the East Kirkton Quarry, uncertainties remain regarding the mechanisms by which dissolved calcium entered the lake. Possible options include leaching from the surrounding volcanic terrain or interactions between the lake’s waters and atmospheric carbon [50,112].

**Fig 10:**
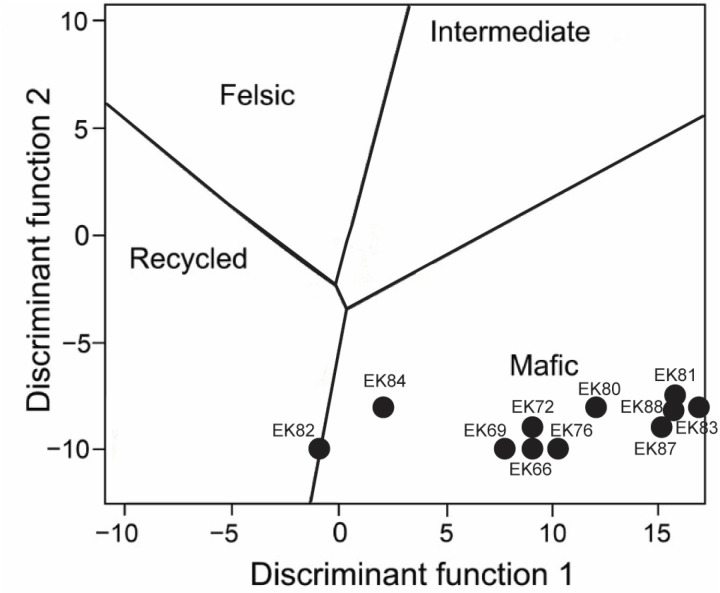
East Kirkton Limestone clastic sediment provenance discrimination diagram based on XRF major oxides elemental composition (after [86]). Function 1= -1.773TiO_2_+0.607Al_2_O_3_+0.76TFe_2_O_3_-1.5MgO+0.616CaO+0.509Na_2_O-1.22K_2_O-9.09. Function 2= 0.445TiO_2_+0.07Al_2_O_3_-0.25TFe_2_O_3_-1.142MgO+0.432Na_2_O+1.426K_2_O-6.861.

In terms of sediment provenance, Fig 10 illustrates that all samples predominantly derive from a mafic source, consistent with [73] conclusions that volcaniclastic sediments played a significant role in shaping the geochemistry of the rocks [10,42,44,51,55]. Sample EK82 represents an exception, plotting along the boundary between a mafic and recycled source. This indicates that the sediment influx to the lake was not exclusively derived from the local volcanic terrain but also included contributions from recycled sediment (Fig 10). An intriguing finding consistent with [73] observations is that samples with higher Al₂O₃ content—associated with shale and/or tuff lithologies—exhibit the highest trace element concentrations (Fig 8, Tables 2–3). This relationship is attributed to the impermeable nature of shales and the adsorption of trace elements by clay minerals [124–126]. Among the eleven samples analyzed in this study, trace element enrichment factor (EF) plots, comparing trace element concentrations to the average upper continental crust values, show enrichment (EF > 1) in elements including V, Cr, Co, Ni, Cu, Zn, Ga, As, Sr, Y, Zr, Nb, Mo, and Ba in most samples (Fig 7, Table 3). This enrichment underscores the influence of both mafic volcaniclastic inputs and hydrothermal processes on the geochemistry of the East Kirkton Limestone.

## Conclusions

This study refines the chronology of the Mississippian tetrapods from the East Kirkton Quarry by presenting the first ^238^U-^206^Pb and ^207^Pb-^206^Pb zircon dates for sample EK82, corresponding to Unit 82 as defined by [46]. These results update previous age interpretations that were based solely on biostratigraphy. Utilizing detrital zircon U-Pb dating and the YMWM maximum depositional age (MDA) approach, we systematically reassess the ages of key taxa from Unit 82, including *Westlothiana lizziae*, *Silvanerpeton miripedes*, *Balanerpeton woodi*, *Ophiderpeton kirktonense*, *Eucritta melanolimnetes*, and *Kirktonecta milnerae*. Our findings establish a refined MDA of 341 ± 3 Ma for Unit 82, situating it within the middle-lower Viséan (Holkerian-Arundian) rather than the previously assigned upper Viséan (Brigantian). However, there is a possibility of a younger age as MDAs are a threshold age limit. This temporal shift repositions the East Kirkton Quarry tetrapod assemblage within the critical temporal framework of Romer’s Gap, effectively bridging a key evolutionary interval in the Mississippian fossil record.

Geochemistry and mineralogical analyses display significant heterogeneity in lithology and mineral composition within the lower East Kirkton Limestone in the East Kirkton Quarry. Variations in CaO, SiO₂, and trace elements such as Ni, Cr, Co, and V reflect episodic contributions of volcanic and detrital sediments, hydrothermal activity, and sporadic marine incursions. The presence of felsic clastic inputs provided sufficient detrital zircons for radiometric dating, underscoring the utility of U-Pb methods in constraining depositional ages within basaltic volcaniclastic settings. Mineralogical analyses indicate that carbonate deposition dominated the restricted hydrothermal lake environment, with episodic clastic influxes introducing quartz, feldspar, and clay minerals. These clastic inputs, coupled with hydrothermal processes, influenced the geochemical signatures, including elevated sulfur concentrations and trace element enrichment. Our findings align with prior studies, confirming that detrital sediment from mafic sources and hydrothermal activity played a significant role in shaping the East Kirkton Limestone’s mineralogy and geochemistry [10,42,44,51,55,73]. The findings of this study provide significant insights into the geological processes that shaped the paleoenvironment of the East Kirkton Quarry. The findings significantly contribute to the understanding of geologic processes influencing the East Kirkton Quarry paleoenvironment, and the refined U-Pb MDA can assist by providing more precise temporal calibrations of phylogenetic models estimating times for the early terrestrial tetrapod lineages.

## Supporting Information

S1 Table Description of the excel file: Detailed U-Pb LA-ICP-MS data. Detailed key and raw data presented under the second tab (Supplementary Material_S1 Table_Garza et al. 2025).
(XLSX)

S2 Table Description of the excel file: Bruker S1 Titan Energy Dispersive XRF Element Detection Limits. Detailed key and instrument detection presented under the second tab (Supplementary Material_S2 Table_Garza et al. 2025).
(XLSX)

S3 Table Description of the excel file: XRF Calibration. Detailed standards key and instrument elemental calibration presented under the second tab (Supplementary Material_S3 Table_Garza et al. 2025).
(XLSX)
